# Supplementary figures and images for: Antibody response to Plasmodium vivax in the context of Epstein-Barr virus (EBV) co-infection: A 14-year follow-up study in the Amazon rainforest
Source: PLoS One. 2025 Jan 29;20(1):e0311704. doi: 10.1371/journal.pone.0311704 (PMC11778755; doi:10.1371/journal.pone.0311704)

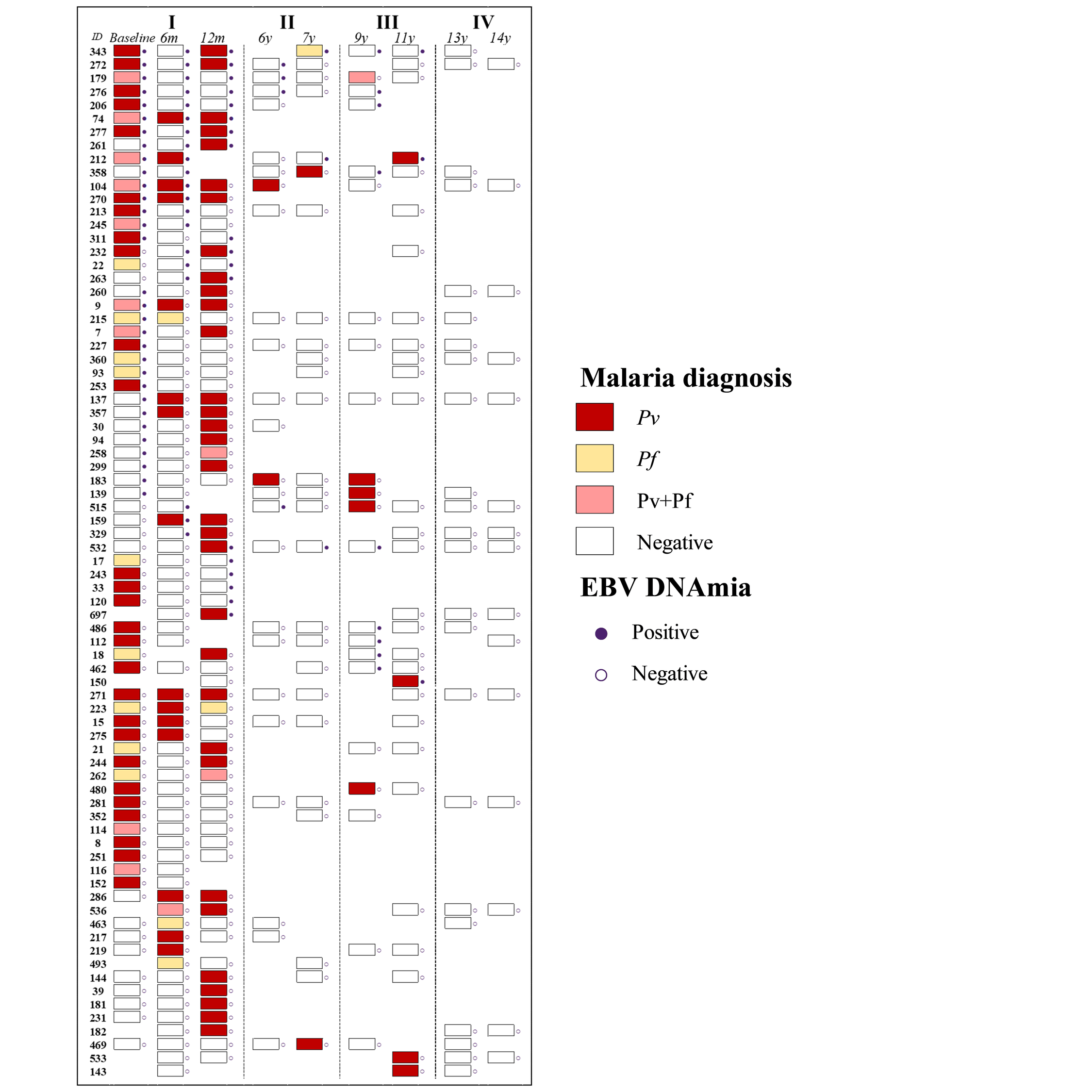

Supplement: S1 Fig — P. vivax infections are represented in red, P. falciparum in yellow, mixed infections (P. falciparum plus P. vivax) in orange, and uninfected samples in white. During the cross-sectional surveys, the presence or absence of episodes of EBV-DNAemia are represented in purple and white circles, respectively. (TIF) [file pone.0311704.s001.tif]

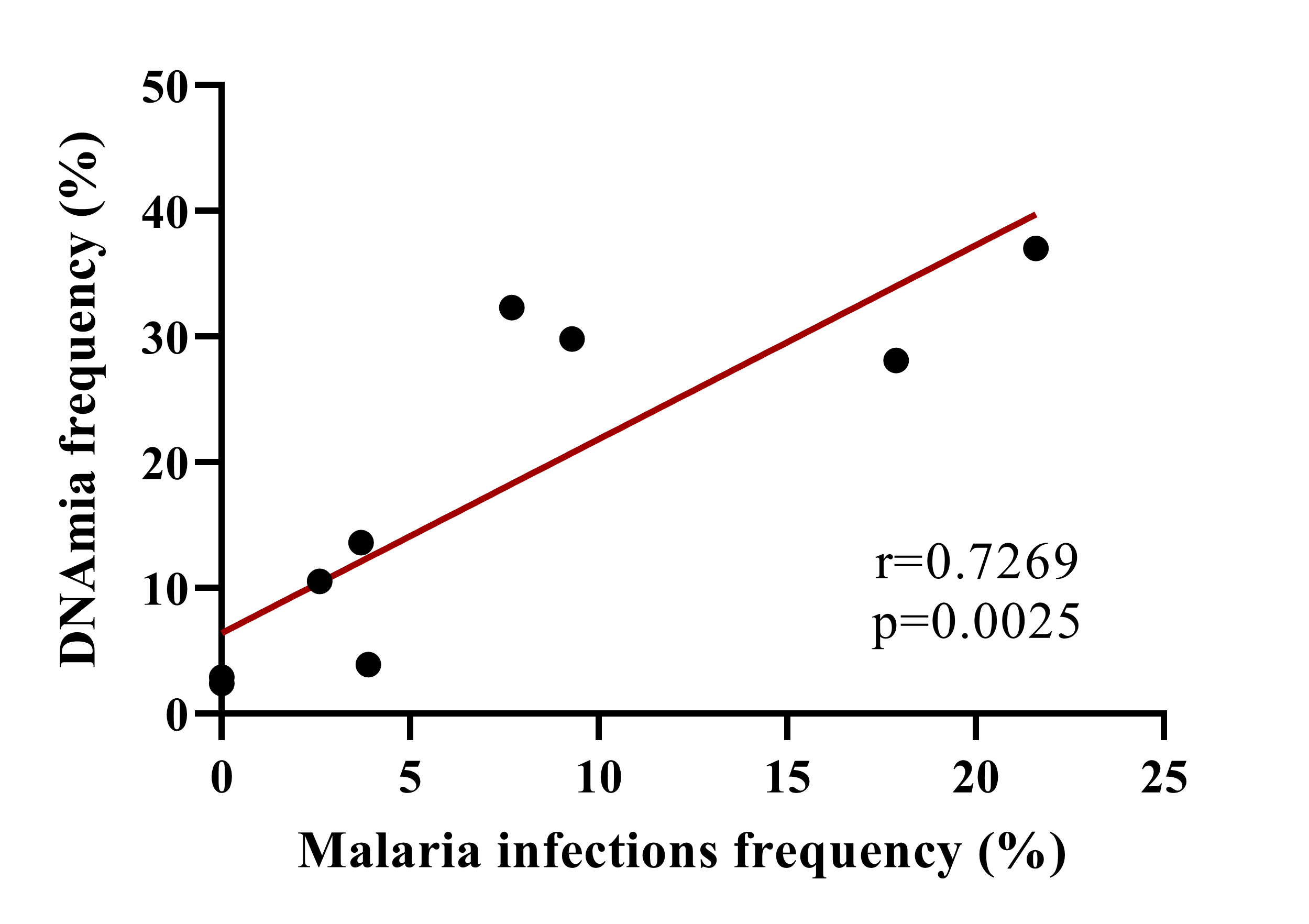

Supplement: S2 Fig — Each dot represents the intersection between the frequency of malaria infections and detectable EBV-DNAemia by cross-sectional survey. (TIF) [file pone.0311704.s002.tif]

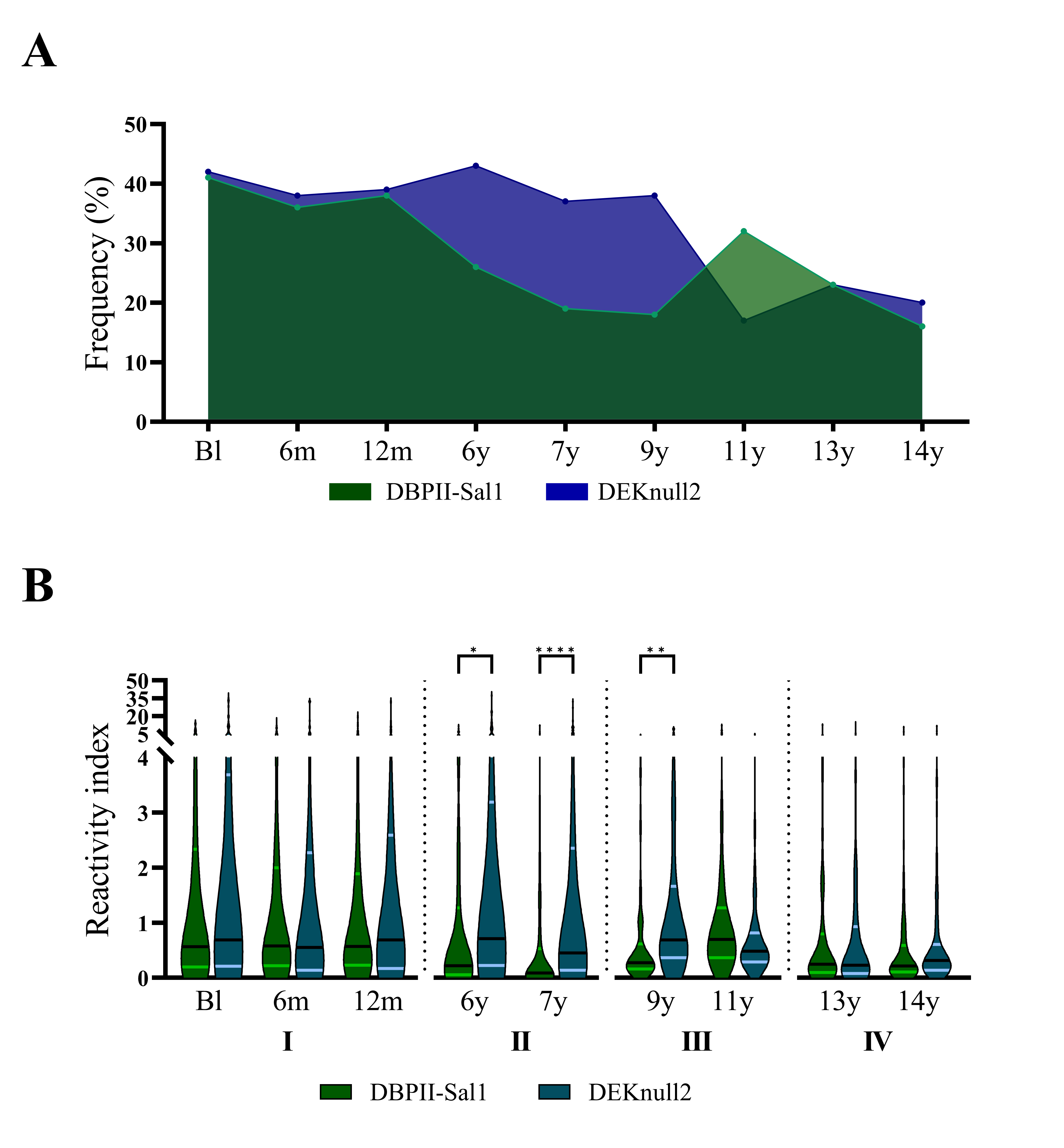

Supplement: S3 Fig — (A) Frequencies and (B) levels of antibodies against DBPII-Sal1 and DEKnull-2, as determined by conventional serological assays (ELISA). Results were expressed as reactivity index (RI), with RI >1.0 considered as an ELISA-positive response. In B, black lines inside the violin plot represent the median RI, with light green and blue lines representing the interquartile range. (TIF) [file pone.0311704.s003.tif]

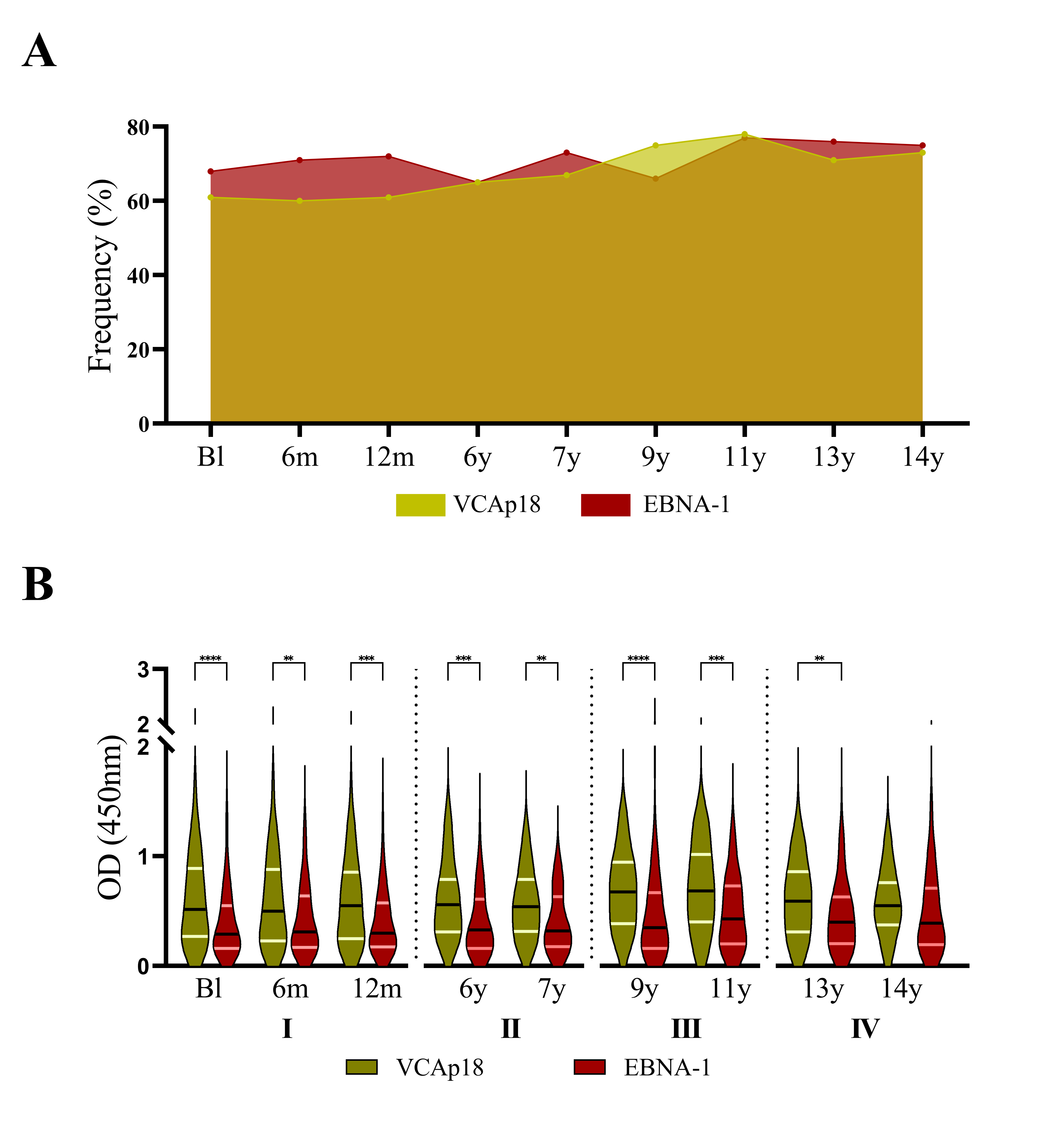

Supplement: S4 Fig — (A) Frequencies and (B) levels of antibodies against VCAp18 and EBNA1, as determined by conventional serological assays (ELISA). Results were expressed as the optical density at 450 nm (OD450), with OD >0.37 and >0.20 considered as ELISA-positive responses to VCAp18 and EBNA1, respectively. In B, black lines inside the violin plot represent the median RI, with yellow and pink lines representing the interquartile range. (TIF) [file pone.0311704.s004.tif]
