# Supplementary material for: Antibody response to Plasmodium vivax in the context of Epstein-Barr virus (EBV) co-infection: A 14-year follow-up study in the Amazon rainforest
Source: PLoS One. 2025 Jan 29;20(1):e0311704. doi: 10.1371/journal.pone.0311704 (PMC11778755; doi:10.1371/journal.pone.0311704)
Supplement: S2 Table — (DOCX) [file pone.0311704.s006.docx]

**S2 Table. DBPII-related antibody responses over time based on the presence or absence of EBV DNAmia**

| **Antigen** | **Characteristics** | **Category** | **Antibody survival analysis**  (average, months)^1^ | **Person-month**^2^ | **Antibody clearance**  n (rate/100 persons/month) | **Relative risk**^3^ | |
| --- | --- | --- | --- | --- | --- | --- | --- |
|  |  |  |  |  |  | **RI (95% CI)** | ***p* value** |
| DBPII-Sal1 | EBV DNAmia episodes | None (n=60) | 156 | 5244 | 27 (0.51) | 1.00 | - |
|  |  | One (n=33) | 168 | 3354 | 19 (0.57) | 0.83 (0.46; 1.50) | 0.546 |
|  |  | Multiple (n=38) | 156 | 4368 | 21 (0.48) | 0.82 (0.46; 1.46) | 0.509 |
|  |  |  |  |  |  |  |  |
| DEKnull2 | EBV DNAmia episodes | None (n=42) | 168 | 4332 | 13 (0.30) | 1.00 | - |
|  |  | One (n=18) | 168 | 1944 | 9 (0.46) | 1.01 (0.42; 2.37) | 0.982 |
|  |  | Multiple (n=23) | 168 | 3012 | 8 (0.27) | 0.83 (0.34; 2.01) | 0.685 |

^1^Time when 50% of individuals lost their antibody response against DBPII-based antigens

^2^Time in months that participants contributed to the follow-up study

^3^Relative risk of antibodies clearance according to episodies of EBV DNAmia
