# Supplementary material for: Antibody response to Plasmodium vivax in the context of Epstein-Barr virus (EBV) co-infection: A 14-year follow-up study in the Amazon rainforest
Source: PLoS One. 2025 Jan 29;20(1):e0311704. doi: 10.1371/journal.pone.0311704 (PMC11778755; doi:10.1371/journal.pone.0311704)
Supplement: S3 Table — (DOCX) [file pone.0311704.s007.docx]

**S3 Table. DBPII-related antibody responses over time based on the antibody response profile against EBV peptides**

| **Antigen** | **Characteristics** | **Category** | **Antibody survival analysis** (average, months)^1^ | **Person-month**^2^ | **Antibody clearance**  n (rate/100 persons/month) | **Relative risk**^3^ | |
| --- | --- | --- | --- | --- | --- | --- | --- |
|  |  |  |  |  |  | **RI (95% CI)** | ***p* value** |
| DBPII-Sal1 | Anti-VCAp18 response profile | NR (n=28) | 156 | 1632 | 8 (0.49) | 1.00 | - |
|  |  | TR (n=30) | 156 | 3768 | 17 (0.45) | 0.61 (0.26; 1.44) | 0.266 |
|  |  | PR (n=73) | 156 | 7566 | 42 (0.55) | 0.83 (0.38; 1.79) | 0.643 |
|  |  |  |  |  |  |  |  |
|  | Anti-EBNA-1 response profile | NR (n=22) | 168 | 1512 | 8 (0.53) | 1.00 | - |
|  |  | TR (n=39) | 168 | 4896 | 24 (0.49) | 0.68 (0.30; 1.52) | 0.356 |
|  |  | PR (n=72) | 156 | 6546 | 35 (0.53) | 1.06 (0.49; 2.29) | 0.879 |
|  |  |  |  |  |  |  |  |
| DEKnull2 | Anti-VCAp18 response profile | NR (n=15) | 168 | 1128 | 3 (0.27) | 1.00 | - |
|  |  | TR (n=20) | 168 | 2646 | 7 (0.26) | 0.81 (0.21; 3.16) | 0.768 |
|  |  | PR (n=48) | 168 | 5514 | 20 (0.36) | 1.18 (0.35; 4.00) | 0.785 |
|  |  |  |  |  |  |  |  |
|  | Anti-EBNA-1 response profile | NR (n=13) | 168 | 828 | 3 (0.36) | 1.00 | - |
|  |  | TR (n=24) | 168 | 3330 | 9 (0.27) | 0.38 (0.10; 1.44) | 0.156 |
|  |  | PR (n=46) | 168 | 5130 | 18 (0.35) | 0.70 (0.20-2.42) | 0.584 |

^1^Time when 50% of individuals lost their antibody response against DBPII-based antigens

^2^Time in months that participants contributed to the follow-up study

^3^Relative risk of antibodies clearance according to antibody reponse profile against VCAp18 and EBNA-1
